# Leveraging primate-specific genomic information for genetic studies of complex diseases

**DOI:** 10.3389/fbinf.2023.1161167

**Published:** 2023-03-28

**Authors:** Wen-Hua Wei, Hui Guo

**Affiliations:** ^1^ Department of Women’s and Children’s Health, Dunedin School of Medicine, University of Otago, Dunedin, New Zealand; ^2^ Department of Biochemistry, University of Otago, Dunedin, New Zealand; ^3^ Centre for Biostatistics, School of Health Sciences, The University of Manchester, Manchester, United Kingdom

**Keywords:** complex disease, colocalization, genetic regulation, pleiotropy, primate-specific genomic information (PSI)

## Abstract

Genomic changes specific to higher primates are regarded as primate-specific genomic information (PSI). Using PSI to inform genetic studies is highly desirable but hampered by three factors: heterogeneity among PSI studies, lack of integrated profiles of the identified PSI elements and dearth of relevant functional information. We report a database of 19,767 PSI elements collated from nine types of brain-related studies, which form 19,473 non-overlapping PSI regions that distribute unevenly but jointly cover only 0.81% of the genome. About 2.5% of the PSI regions colocalized with variants identified in genome-wide association studies, with disease loci more likely colocalized than quantitative trait loci (*p* = 1.6 × 10^−5^), particularly in regions without obvious regulatory roles. We further showed an *LRP4* exemplar region with PSI elements orchestrated with common and rare disease variants and other functional elements. Our results render PSI elements as a valuable source to inform genetic studies of complex diseases.

## 1 Introduction

Using human-specific genomic changes to inform studying human-specific features has been a hot topic (Pollen et al., 2023) since the first discovery of human accelerated regions (HARs) that were highly conserved across vertebrates but with an accelerated rate of evolution from chimpanzees to humans in nucleotide substitution (Pollard et al., 2006). The roles of HARs have been confirmed in regulation of human-specific morphological features such as limb and brain development (Prabhakar et al., 2008; Boyd et al., 2015). Additional human-specific genomic changes have been uncovered including human accelerated DNase I hypersensitive sites (haDHS) (Gittelman et al., 2015), human biased cis-regulatory elements (Prescott et al., 2015), Hominin-specific gene regulatory elements (hsGRE) (Castelijns et al., 2020), and large structural changes such as human-specific insertions (hsInsert) that tend to be fixed in human populations (McLean et al., 2011; Kronenberg et al., 2018).

Furthermore, researchers have uncovered substantial genomic changes that are evolutionarily specific to primates (Tay et al., 2009; Vermunt et al., 2016). These primate-specific genomic information (PSI), including the human-specific genomic changes, form an important resource that could potentially shed light on complex mechanisms regulating human phenotypes (Juan et al., 2023). For example, inactivation of the uricase gene in higher primates has led to a much higher base level of urate concentration than that in species with intact uricase such as mice, and thus may have shaped a special urate-mediated physiology (Lu et al., 2019). Indeed, PSI elements were found in regulatory regions of urate associated genes (Takei et al., 2021) and in the human uricase gene *UOX* (Castelijns et al., 2020).

There are however a few issues limiting PSI applications. First, heterogeneity among PSI elements identified in different studies using evolved definitions and small samples. For example, overlaps exist in only a few of ∼2,700 HARs reported from various studies (Levchenko et al., 2018). Second, there is dearth of functional information for the vast majority of the PSI elements, which often requires right tissues or cell types to dissect (Doan et al., 2016; Parikshak et al., 2016; Won et al., 2019) and thus is rather expensive and time consuming. Third, a clear profile of the PSI elements and their interrelations is lacking, e.g., genomic distribution, frequencies in populations.

Another difficulty is to clarify potential roles of PSI elements in regulating human diseases, given that PSI elements are concerned at the species level rather than within-species or individual levels. PSI elements tend to be subject to positive selection leading to fixation in human populations (McLean et al., 2011; Kronenberg et al., 2018) while such positive selection could be reverted to negative selection during human evolution (e.g., adaptation to different environments) (Doan et al., 2016; Levchenko et al., 2018), leading to phenotypic variations including diseases. Furthermore, mutations can happen in PSI elements where deleterious variants may be associated with diseases such as neural disorders underlying the emergence of the human brain (Doan et al., 2016; Castelijns et al., 2020; Takei et al., 2021). These attributes together render PSI elements as favorable targets for new insights into disease associations and primate-specific regulatory mechanisms.

Here we characterize PSI elements collated from a range of publications mostly on brain-related features and their colocalization with common variants identified in genome-wide association studies (GWASs). We quote genomic positions in GRCh37/hg19 throughout.

## 2 Materials and methods

To prepare for a whole genome sequencing based project aimed to define human specific genetic variants in brain developmental disorders, journal articles studying primate-specific and/or human-specific genomic features were reviewed and selected based on relevance and usefulness for annotating sequencing variants. We then extracted data from each selected journal article and converted the genome coordinates to GRCh37/hg19 when necessary.

The PSI elements collated were first sorted by chromosome locations and merged into PSI regions without overlaps with each other (Supplementary Table S1). Subsequently, distribution and summary statistics of PSI regions were generated. The PSI regions were then used as a map to examine colocalization of common variants identified in GWASs of 10 quantitative traits and 15 dichotomous disease traits outlined in Supplementary Table S2. Mapping GWAS variants to PSI regions and statistical tests were performed using R (R Core Team. R, 2018) (https://www.R-project.org, v4.1.1). Chi-square tests were used to examine hypothesis that no difference between the expected and the observed frequencies in one or more categories of a contingency table, or hypothesis of independence between two distributions. For each GWAS, genome-wide significant (*p* < 5 × 10^−8^ or log_10_Bayes Factor >6 as appropriate in the study) variants were extracted and each tested for mapping to the PSI regions and recorded with the PSI type if mapped using a self-developed R script (available on request). Then the numbers of independent loci were derived from the input and the mapped variants respectively by counting linkage disequilibrium blocks (*r* ^2^ > 0.8) associated with the lead associated variants that were mutually independent and reported in the original paper. For example, if five mapped variants are within a linkage disequilibrium block of a GWAS lead associated variant, they will be counted as one mapped locus. Mapping rate was then calculated as percentage of the number of mapped loci out of the number of GWAS loci. The GTEx Portal (Lonsdale et al., 2013) (https://gtexportal.org/home/) was used to retrieve functional information such as expression quantitative trait loci (eQTL) or splicing quantitative trait loci (sQTL) for variants of interest.

## 3 Results

**FIGURE 1:**
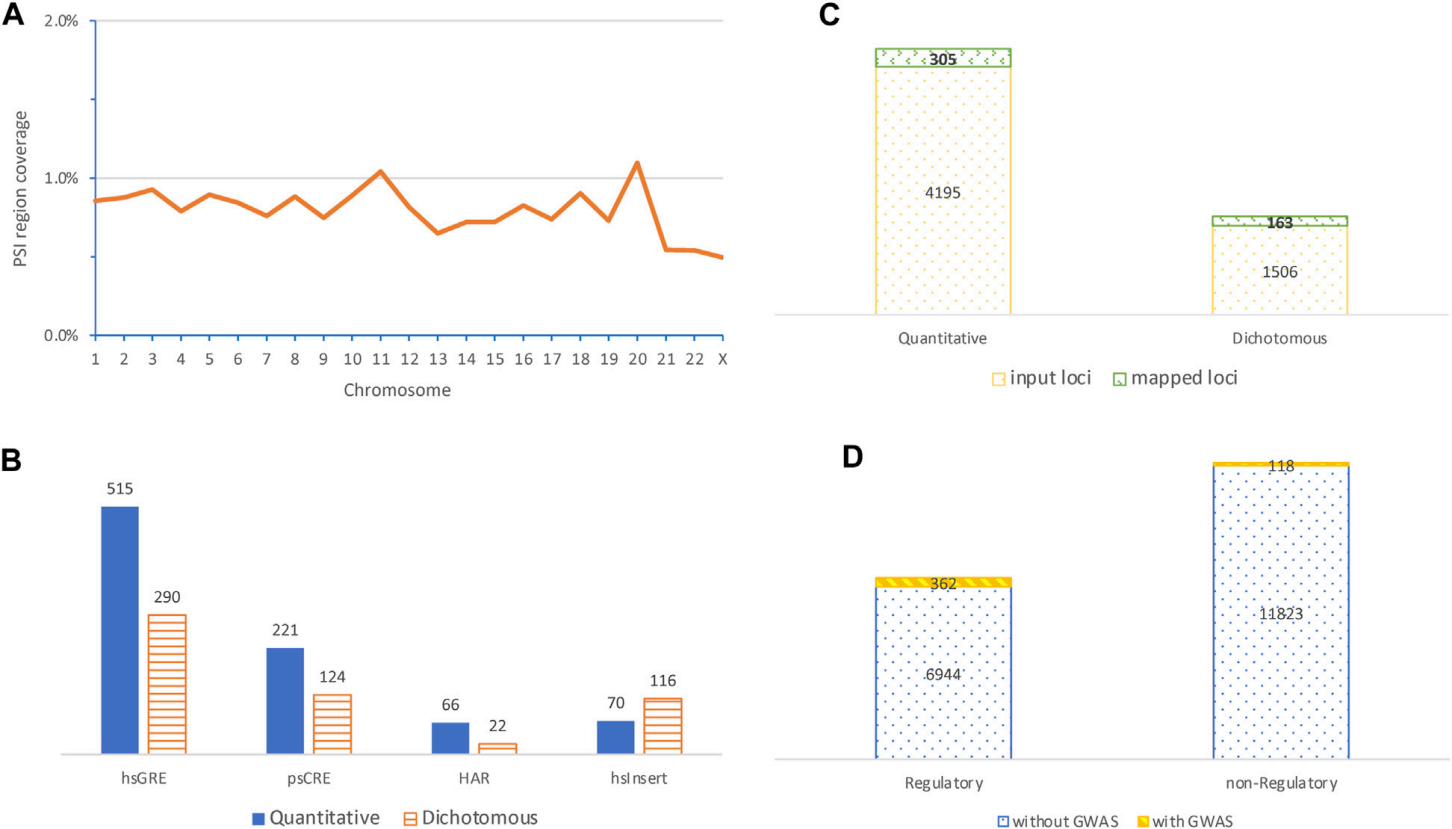
Characteristics of PSI regions and their colocalization with variants identified in genome-wide association studies. **(A)** Distribution of chromosomal coverage of PSI regions (top left); **(B)** contrast of the number of mapped GWAS variants identified in quantitative traits with that from dichotomous diseases by four mostly mapped PSI types (bottom left); **(C)** contrast of the number of input GWAS loci with the number of loci mapped to PSI regions by categories of quantitative or dichotomous phenotypes (top right); **(D)** comparison of the number of PSI regions with no GWAS loci mapped to against that with mapped GWAS loci by categories of PSI regions with or without obvious regulatory roles (bottom right, PSI regions with multiple PSI types were excluded for simplicity).

We studied 19,767 PSI elements identified from nine studies each with a different PSI type based on DNA sequences and/or brain related tissues or cell types (Table 1). These PSI elements formed 19,473 non-overlapping PSI regions that are generally small (Supplementary Table S1), with an average length of 1,268 base pairs, together cover only 0.81% of the genome. Only 226 PSI regions (1.2%) were derived from multiple PSI elements confirming fairly limited overlaps between PSI types. The PSI regions distributed unevenly by chromosome (*p* < 2.2 × 10^−16^, Chi-squared test accounting for chromosomal lengths), where the chromosomal length coverage by PSI regions fluctuated around 0.8% but was above 1% in chromosomes 11 and 20 and as low as 0.5% in chromosomes 21, 22, and X (Figure 1A).

**TABLE 1:** Summary information of the PSI elements^a^.

PSI_type	PSI_description	Brief	Number	Length% (%)	References
1	human accelerated region	HAR	2737	2.8	Doan et al. (2016)
2	human gained enhancers during corticogenesis	HGE	63	0.1	Reilly et al. (2015)
3	human-biased cis-regulatory elements in cranial neural crest cells	hbCRE	1000	0.8	Prescott et al. (2015)
4	primate-specific cis-regulatory elements in brain	psCRE	1488	19.9	Vermunt et al. (2016)
5	recurrent *de novo* mutations in regulatory elements of neurodevelopment	DNM	31	0.1	Short et al. (2018)
6	human-specific insertion	hsInsert	11874	41.7	Kronenberg et al. (2018)
7	primate-specific transcriptional unit	psTU	131	1.8	Tay et al. (2009)
8	human accelerated DNase I hypersensitive site	haDHS	524	0.7	Gittelman et al. (2015)
9	Hominin-specific gene regulatory element	hsGRE	1919	32.6	Castelijns et al. (2020)

^a^ Length%: percentage of the total length per PSI, type over the total length of the PSI, regions (24,708,805 base pairs); brief: Brief name of PSI_type; ref: Reference of the original study.

Of the 19,473 PSI regions, 493 (∼2.5%) were mapped by 1,499 (representing 468 independent loci) out of 253,200 genome-wide significant variants (representing 5,701 independent loci) identified in GWASs of 10 quantitative and 15 dichotomous disease traits, giving a mapping rate of 0.6% (or 8.2%) at the variant (or locus) level (Supplementary Table S2). Of the 1,499 mapped variants, 332 (∼22%) were pleiotropic and associated with multiple traits (Supplementary Table S3). At the variant level, the top mapped PSI types were hsGRE, psCRE, hsInsert, and HAR (Figure 1B), where hsInsert was the only one mapped with more disease variants than quantitative trait variants and was significantly different from the remaining (*p* = 9.3 × 10^−12^, Chi-squared test). At the locus level, disease loci were more likely mapped to PSI regions than quantitative trait loci (*p* = 1.6 × 10^−05^, Chi-squared test) (Figure 1C). Considering hsInsert and psTU as the only PSI types without obvious regulatory evidence, a further locus level comparison suggested that the mapped GWAS loci were highly biased towards regulatory regions (*p* = 1.4 × 10^−66^, Chi-squared test) (Figure 1D). Nevertheless, non-regulatory PSI regions colocalized with 23.9% (118 out of the 493) of the mapped GWAS loci and thus played an important part as well.

**FIGURE 2:**
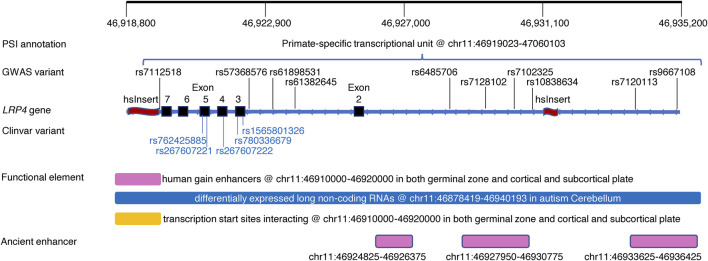
An exemplar region within the *LRP4* gene harboring common and rare pathogenic variants, PSI and other regulatory elements. Top track: Genomic positions of the region of interest; PSI annotation track: Alignment of a PSI element; GWAS variants track: Alignment of genome-wide significant variants associated with Schizophrenia and/or body mass index (Supplementary Tables S2, S3); *LRP4* gene track: Refseq gene *LRP4* marked with exons (black rectangle) and two human-specific insertions (red wave) within the region of interest; Clinvar variant track: Alignment of Clinvar pathogenic variants causing Cenani-Lenz syndactyly syndrome (https://www.ncbi.nlm.nih.gov/clinvar/); Functional element track: Alignment of human gain enhancer (Won et al., 2019), long non-coding RNA (Parikshak et al., 2016), interacting transcription start starts (Won et al., 2019); Ancient enhancer track: Alignment of ancient enhancers in neocortex shared across mammals (Emera et al., 2016).

Figure 2 showed an exemplar region within the *LRP4* gene harboring GWAS variants and rare pathogenic variants as well as a psTU overlapped with a hsInsert downstream and another hsInsert upstream. The region also harbors brain related functional elements including human gain enhancers (Reilly et al., 2015), differentially expressed long non-coding RNA (Parikshak et al., 2016), interacting transcription start sites (Won et al., 2016), and three ancient enhancers in neocortex shared across mammals (Emera et al., 2016).

## 4 Discussion

We presented a profile of PSI elements distributed across the genome for the first time. These PSI elements jointly covered only 0.81% of the genome space but colocalized with GWAS common variants in 2.5% of the non-overlapping PSI regions derived. Disease variants appeared to be more likely to locate in a PSI region than quantitative trait variants, particularly in those without obvious regulatory roles (Figure 1). We further showed in the *LRP4* exemplar region the colocalization of PSI elements with common and rare variants as well as additional functional elements (Figure 2) signaling complex regulatory mechanisms. These results together render PSI elements as a valuable source to inform genetic studies of complex diseases.

It is not unexpected that majority of the mapped PSI regions are regulatory because GWAS variants tend to appear in regulatory regions (Edwards et al., 2013; Buniello et al., 2019). However, since PSI regulatory elements conveying evolution perspectives, they can bring new insights into complex regulation mechanisms as shown in the *LRP4* example here and previous examples of *SLC2A9* regulating urate levels (Takei et al., 2021) and *CACNA1C* regulating Bipolar Disorder and Schizophrenia (Song et al., 2018). On the other hand, non-regulatory PSI regions are clearly involved in various phenotypes particularly diseases. Considering the downstream hsInsert in the *LRP4* example (Figure 2), within it we did find rs186930464, instead of being fixed in human genome (Kronenberg et al., 2018), with varied allele frequencies in the 1,000 Genomes project and strong eQTLs and sQTLs in multiple tissues (https://gtexportal.org/home/snp/rs186930464) (Supplementary Tables S4, S5). Integrating existing functional information of its overlapped regulatory elements could better our understanding of the hsInsert and other PSI elements that were often derived from small samples with limited replication. Further functional investigation is needed to understand how these “non-regulatory” PSI elements work in shaping disease phenotypes.

The observed colocalization of PSI regions with GWAS common variants can be regarded as new evidence supporting the hypothesis of speciation characterized by substantial rewiring of the regulatory circuitry of functional genes (Castelijns et al., 2020), suggesting that every human trait would have some human-specific characteristics, as previously showed in immune and metabolic conditions (O’Bleness et al., 2012). Since PSI elements are shared across human populations, they are likely associated with common diseases but unlikely with rare diseases.

This pilot study is limited by incomprehensive inclusion of PSI elements and incomprehensive analysis scenarios. Caution is therefore recommended when interpreting the results. Further investigations are warranted to comprehensively explore PSI values in informing genetic studies of complex diseases.

## Data Availability

The original contributions presented in the study are included in the article/Supplementary Material, further inquiries can be directed to the corresponding author.
